# *Clostridium botulinum* C3bot mediated effects on cytokine-induced psoriasis-like phenotype in full-thickness skin model

**DOI:** 10.1007/s00210-023-02718-9

**Published:** 2023-09-14

**Authors:** Astrid Rohrbeck, Vanessa Anna Bruhn, Nali Hussein, Sandra Hagemann, Ingo Just

**Affiliations:** Institute of Toxicology, Hannover Medical School, Carl-Neuberg-Str. 1, D-30625 Hannover, Germany

**Keywords:** C3 exoenzyme, ADP-ribosyltransferase, Psoriasis-like phenotype, Claudin-1, Full-thickness skin model, IL-6

## Abstract

*Clostridium botulinum* C3 exoenzyme (C3bot) exclusively inhibits RhoA, B and C by ADP-ribosylation and is therefore used as a cell-permeable tool for investigating the cellular role of these Rho-GTPases. Rho-GTPases represent a molecular switch integrating different receptor signalling to downstream cascades including transcriptional cascades that regulate various cellular processes, such as regulation of actin cytoskeleton and cell proliferation. C3bot-induced inhibition of RhoA leads to reorganization of the actin cytoskeleton, morphological changes, and inhibition of cell proliferation as well as modulation of inflammatory response. In this study, we characterized the C3bot-mediated effects on a full-thickness skin model exhibiting a psoriasis-like phenotype through the addition of cytokines. Indeed, after the addition of cytokines, a decrease in epidermal thickness, parakeratosis, and induction of IL-6 was detected. In the next step, it was studied whether C3bot caused a reduction in the cytokine-induced psoriasis-like phenotypes. Basal addition of C3bot after cytokine induction of the full-thickness skin models caused less epidermal thinning and reduced IL-6 abundance. Simultaneous basal incubation with cytokines and C3bot, IL-6 abundance was inhibited, but epidermal thickness was only moderately affected. When C3bot was added apically to the skin model, IL-6 abundance was reduced, but no further effects on the psoriasis-like phenotype of the epidermis were observed. In summary, C3bot inhibits the cytokine-induced expression of IL-6 and thus may have an impact on the pro-inflammatory immune response in the psoriasis-like phenotype.

## Introduction

The skin forms the interface between the human body and the environment. It protects our body against various biological and chemical hazards and from desiccation in a dry environment. The skin consists of three layers, from the superficial to the innermost layer: the epidermis, the dermis, and the hypodermis. The epidermis consists of various layers, of which the *stratum corneum* is the uppermost nonviable layer (Kanitakis 2002). During the formation of the *stratum corneum*, keratinocytes move into the direction of the skin surface. In the *stratum basale* the keratinocytes proliferate. As soon as the cells escape from *stratum basale*, keratinocyte differentiation is initiated. When keratinocytes enter the *stratum granulosum*, the cornification proteins including (pro-)filaggrin, proline-rich proteins, involucrin, and loricrin are synthesized (Steven and Steinert 1994; Shamilov et al. 2021). When passing the viable epidermis-*stratum corneum* interface, the cells transform into dead flattened cells (corneocytes). The corneocytes are embedded in a lipid matrix as “bricks in mortar.” The lipids in the intercellular regions form crystalline lipid lamellae (Hill and Wertz 2003). The corneocytes and the lipid lamellae are oriented approximately parallel to the skin surface. The lipid organization in *stratum corneum* plays an important role in the barrier function of the skin (van Smeden and Bouwstra 2016). Because of the highly impermeable character of the cornified envelope, compounds applied onto the skin are considered to penetrate primarily via the transepidermal route into the deeper regions of the skin. Thus, improving cellular uptake is important for therapeutic application. It is reported in the literature that 1–5% DMSO (dimethyl sulfoxide) accelerates the uptake of substances into the cells (Wang et al. 2016). Therefore, DMSO is used as a pharmaceutical penetration enhancer to facilitate transdermal drug delivery (Karrie Marren 2011). In the current study, we used Phenion^®^ 3D full-thickness skin model (Henkel, Germany). These commercial skin model was further improved and resulted in the generation of 3D cultures with a higher degree of differentiation, a better morphology and the presence of lamellar bodies that extruded their content at the interface between *stratum granulosum* and *stratum corneum*. This skin model shows similar epidermal morphology comparable to native human skin (Ackermann et al. 2010). Additionally, this skin model mimics the route of exposure for dermally applied chemicals and therefore allows for testing conditions closer to the intended situation of use. Moreover, inflammatory stimuli could drive the development of the psoriasis-like phenotype in this model (Singh et al. 2020). Psoriasis is a chronic inflammatory disease characterized by prominent epidermal proliferation and keratinocyte hyperplasia with altered differentiation and scale production. The epidermis becomes thick with overgrowth of the *stratum corneum* containing immature keratinocytes and alternated expression of tight junction proteins like claudin-1 and zonula occludens-1 (Yoshida et al. 2001; Pummi et al. 2001). A large number of cytokines and chemokines have been proposed to contribute to the pathogenesis of psoriasis (Nickoloff et al. 2007). In this context, an increased transcript level of pro-inflammatory factors such as IL-6 is the essential hallmark of the disease (Grossman et al. 1989; Fujishima et al. 2010; Johnston et al. 2013) in addition to further described cytokines important in psoriasis pathogenesis such as TNF-alpha, IL-22 and IL-17 (Zheng et al. 2007; Nickoloff 2007). Interestingly, incubation of 3D full-thickness skin models with recombinant human IL-17A, IL-22 and TNF-alpha for 5 days leads to hyperkeratosis and parakeratosis, both characteristic of psoriasis (Clarysse et al. 2019).

*Clostridium botulinum* C3 exoenzyme (C3bot) showed significant effects on axon growth in animal models and in primary cells (Ahnert-Hilger et al. 2004; Loske et al. 2012). The C3 exoenzyme is an ADP-ribosyltransferase, which was first described in *Clostridium botulinum* (Aktories et al. 1988; Rubin et al. 1988). With the enzymatic domain, C3bot is able to cleave the ubiquitous nicotinamide adenine dinucleotide (NAD^+^) into nicotinamide and ADP-ribose and to transfer ADP-ribose on Rho GTPases RhoA, B and C in a next step (Sekine et al. 1989). Incubation of microglia with C3bot induced a dose-dependent increase in the production of TNF-alpha and IL-6 (Hoffmann et al. 2008). As already mentioned, these two cytokines also play a major role in psoriasis. Therefore, in this study, we focused on the impact of C3bot on the cytokine-induced psoriasis-like phenotype.

## Materials and methods

### Skin tissues

Phenion^®^ full-thickness skin models were purchased from Henkel (Phenion^®^FT; Düsseldorf, Germany; www.phenion.com) Pleaand cultured in small Petri dishes (3.5 cm in diameter) filled with 5 -ml pre-warmed air-liquid-interface (ALI) medium. The ALI medium, which was provided by the manufacturer, lacked phenol red and was refreshed one time after an initial overnight equilibration period. The skin models were subjected to experiments after the overnight equilibration at 37 °C and 5% CO_2_.

### Experimental design

The study consists of three experimental approaches. In the first part, skin models were exposed basally with cytokines (rh-IL-17A (10 ng/ml), rh-IL-22 (25 ng/ml), rh-TNF-alpha (10 ng/ml); PeproTech, Hamburg, Germany) for 5 days to induce psoriasis-like phenotype. These skin models were then basally incubated with 50 nM of C3bot for 3 days. In the second part, the skin models were basally treated with the cytokines and C3bot for a total of 8 days. In the last part, the skin models were treated basally with the cytokines for 5 days, and then 50 nM of C3bot was added apically for 3 days. In all three experimental batches, the medium and C3bot were changed every 48 h. In addition to the treated skin models, a negative control of untreated tissues or a solvent control was also included in all experimental approaches. Each control and treatment group was tested independently three times. C3bot and 1% DMSO were applied topically in a volume of 12 µl for 72 h. In this third set of experiments, 1% DMSO was added apically to each skin model (untreated control means here 1% DMSO but no cytokines or C3bot treatment). At 8 days, the skin models were opportunely cut with a sterile blade in different sections, snap frozen on dry ice and stored at  − 80 °C for successive experiments, in particular Western blot analysis, H&E staining, and immunocytochemistry.

### Expression and purification of recombinant C3bot proteins

C3bot wild type was expressed as recombinant GST-fusion proteins in *E. coli* TG1 harboring the respective DNA fragment (gene of *Clostridium botulinum* C3, accession no. X59039) in the plasmid pGEX-2 T (GE Healthcare Europe GmbH, Freiburg, Germany) as described previously (Höltje et al. 2005). Briefly, pGEX-2 T plasmids encoding wild-type C3bot were transformed into *E. coli* TG1 cells. Starter cultures grown in Luria-Bertani broth with ampicillin at 37 °C overnight were diluted into fresh media. Cells were grown for 3 h (OD_600_ = 0.7) at 37 °C, and IPTG was added to a final concentration of 200 µM to induce the C3bot expression for another 3 h. Cells were harvested by centrifugation, resuspended in lysis buffer (20 mM Tris/HCl (pH7.4), 10 mM NaCl, 5 mM MgCl_2_, 5 mM DTT, and 1 mM PMSF) and lysed by ultrasonic (3 × 20 s, 90% cycle, 20% power) on ice. The lysate was centrifuged for 30 min at 15,000 × g. The supernatant was incubated with glutathione-sepharose beads for 5 h at 4 °C to bind the GST-fusion protein. Beads were washed with buffer containing 50 mM Tris/HCl (pH 8.0), 10 mM Glutathione, 100 mM NaCl, and PMSF. The GST-fused C3bot protein was cleaved from the beads with thrombin (Sigma-Aldrich, Chemie GmbH, Munich, Germany) in a buffer containing 50 mM Tris/HCl (pH 8.0), 50 mM NaCl, and 2.5 mM CaCl_2_. Purified proteins were then concentrated to 1 ml with a Centricon microconcentrator (Amicon, Danvers, MA, USA) with a 30,000 molecular weight cutoff. Thrombin was removed from purified C3bot by use of p-amino-benzamidine-beads (Sigma-Aldrich, Chemie GmbH, Munich, Germany). Buffer exchange was performed by the use of PD 10 column (GE Healthcare), and purified C3bot was eluted in 20 mM HEPES (pH 7.5). Eluted proteins were analyzed by 15% SDS-PAGE and stained with Coomassie blue. ADP-ribosyltransferase activity was measured by an in vitro ADP-ribosylation assay.

### H&E staining

One-half of the frozen skin model was transversely mounted on a cryostat chuck using a cryo-embedding medium (Tissue-Tek^®^ O.C.T.TM) and frozen on the cryostat. The tissues were then cut with a cryo-microtome (Kryotom Leica CM1900UV) in 10-µm thick slices for the H&E staining. Sections were dried at room temperature and fixed in cold acetone. The acetone (Sigma-Aldrich Chemie GmbH, Munich, Germany) fixed, air-dried, slices were stained in hematoxylin solution (Sigma-Aldrich Chemie GmbH, Munich, Germany) for approximately 5 min. The slides were then washed and rinsed in tapwater until the color changes, from red to blue. After completion of this “blueing” process, the slides were stained in eosin g solution (Sigma-Aldrich Chemie GmbH, Munich, Germany) for approximately 3 s. They were then rinsed in water and dehydrated with increasing ethanol concentration (until 95% v/v). The slides were then cleared in xylene (Sigma-Aldrich Chemie GmbH, Munich, Germany) and mounted using Entellan (Merck-Millipore). One trained pathologist (Christopher Werlein, Institute of Pathology, MHH) examined the stained tissue sections and selected the technically best section for the measuring procedure. Images were acquired using a Zeiss AxioObserver 5 microscope system (20 × objective, Carl Zeiss AG, Oberkochen, Germany). Three images were acquired for each sample. For each skin model, three measurements were taken from different fields of view for each of the three individual sections analyzed (9 total measurements/skin model). The thickness of *stratum granulosum* to *stratum basale* (from the upper part of the granular layer to the bottom of the epidermis) was measured using a calibrated scale bar with Zeiss Zen 3.1 software (Carl Zeiss AG, Oberkochen, Germany). Total epidermal thickness cannot be determined because the *stratum corneum* is often detached.

### Immunohistochemistry

From all skin models, 10-µm cryostat sections were prepared and subsequently fixed in 4% formaldehyde in phosphate-buffered saline (PBS) (pH 7.4) at room temperature for 15 min. Fixed slices were then washed and permeabilized with 0.3% (*w/v*) Triton X-100 in PBS supplemented with 5% BSA. Claudin-1 was stained by α-claudin-1 rabbit polyclonal antibody (2 mg/ml in 1% BSA, PA5-16833, Cell Signaling Technology) and zonula occludens-1 by use of α-zonula occludens-1 mouse monoclonal antibody (1:100 in 5% BSA, ZO1-1A12, Thermo Fischer Scientific Inc., Rockford, IL, USA) and Alexa 488-conjugated secondary antibody (Life Technologies GmbH, Darmstadt, Germany) for 1 h at room temperature. Then, a 0.1 µg/ml solution of DAPI (Serva Electrophoresis GmbH, Heidelberg, Germany) in phosphate-buffered saline supplemented with 0.1% (*w/v*) Tween-20 was used for nuclei staining for 15 min at 37 °C. Lastly, sections were washed three times in PBS and mounted in prolong Antifade (Thermo Fischer Scientific Inc., Rockford, IL, USA). Images were acquired using a Zeiss AxioObserver5 microscope system (40 × and 63 × oil immersion objective, Carl Zeiss AG, Oberkochen, Germany). Fluorescent dyes were excited at a wavelength of 491 nm (green fluorescence) and 405 nm (blue fluorescence), respectively. All parameters (laser percentage and voltage, light, gain, exposure, and offset values) remained constant during image acquisition. Three images were acquired for each sample. The images were converted to JPEG files and ImageJ (version 1.53e) was chosen for the quantification of the mean fluorescence of three rectangle region of interests (ROIs) per image (9 total measurements/skin model).

### Western blot analysis

Frozen tissues were thawed in freshly prepared cold lysis buffer containing: 20 mM Tris/HCl (pH7.4), 10 mM NaCl, 5 mM MgCl_2_, 5 mM DTT, and 1 mM PMSF and lysed by ultrasonic (3 × 20 s, 90% cycle, 20% power) on ice using a sonotrode (Bandelin Electronic, Berlin, Germany). Lysates were centrifuged at 2000 g for 10 min at 4 °C. Samples were then heated to 95 °C for 5 min. Complete lysate proteins were separated using sodium dodecylsulfate polyacrylamide gel electrophoresis (SDS-PAGE) (Cti*-*Chemie *u.* Werkstoff-Technik GmbH, Idstein, Germany) and subsequently transferred onto nitrocellulose membranes by a tank blot system. The membranes were blocked with 5% (*w/v*) nonfat dried milk for 60 min; incubation with primary antibody was conducted overnight at 4 °C and treatment with the secondary antibody at room temperature for 1 h. For Western blot analysis, the following primary antibodies were used: IL-6 was identified using a rabbit monoclonal IgG from Invitrogen (AB_253252, Invitrogen). ADP-ribosylated RhoA was detected by a specific antibody against ADP-ribosylated RhoA (ViF140_A1-hFc antibody was kindly provided by Viola Fühner and Michael Hust, Technische Universität Braunschweig, Germany (Rohrbeck et al. 2016)). Actin (Sigma-Aldrich Chemie GmbH, Munich, Germany) was used as the loading control. For the chemiluminescence reaction, electrochemiluminescence (ECL) Femto (Pierce, Thermo Fisher Scientific Inc., Rockford, IL, USA) was used. Chemiluminescent signals were detected using an Intas Chemostar ECL imager (Intas, Göttingen, Germany). All signals were analyzed densitometrically using LabImage 1D software (Intas, Göttingen, Germany) and normalized to β-actin signals.

### Statistical analysis

All experiments were performed independently three times. Results from representative experiments are shown in the figures. Graphs and statistical significance were calculated with GraphPad Prism software (v. 8, GraphPad Software, Inc., San Diego, CA). Data were examined using mixed analysis of variance (ANOVA) followed by post hoc Bonferroni correction. Differences were considered to be statistically significant at *p* ≤ 0.05 (*p ≤ 0.05, **p ≤ 0.01, and ***p ≤ 0.001).

## Results

### Cytokines induce psoriasis-like changes in Phenion^®^ full-thickness skin models

**Fig. 1:**
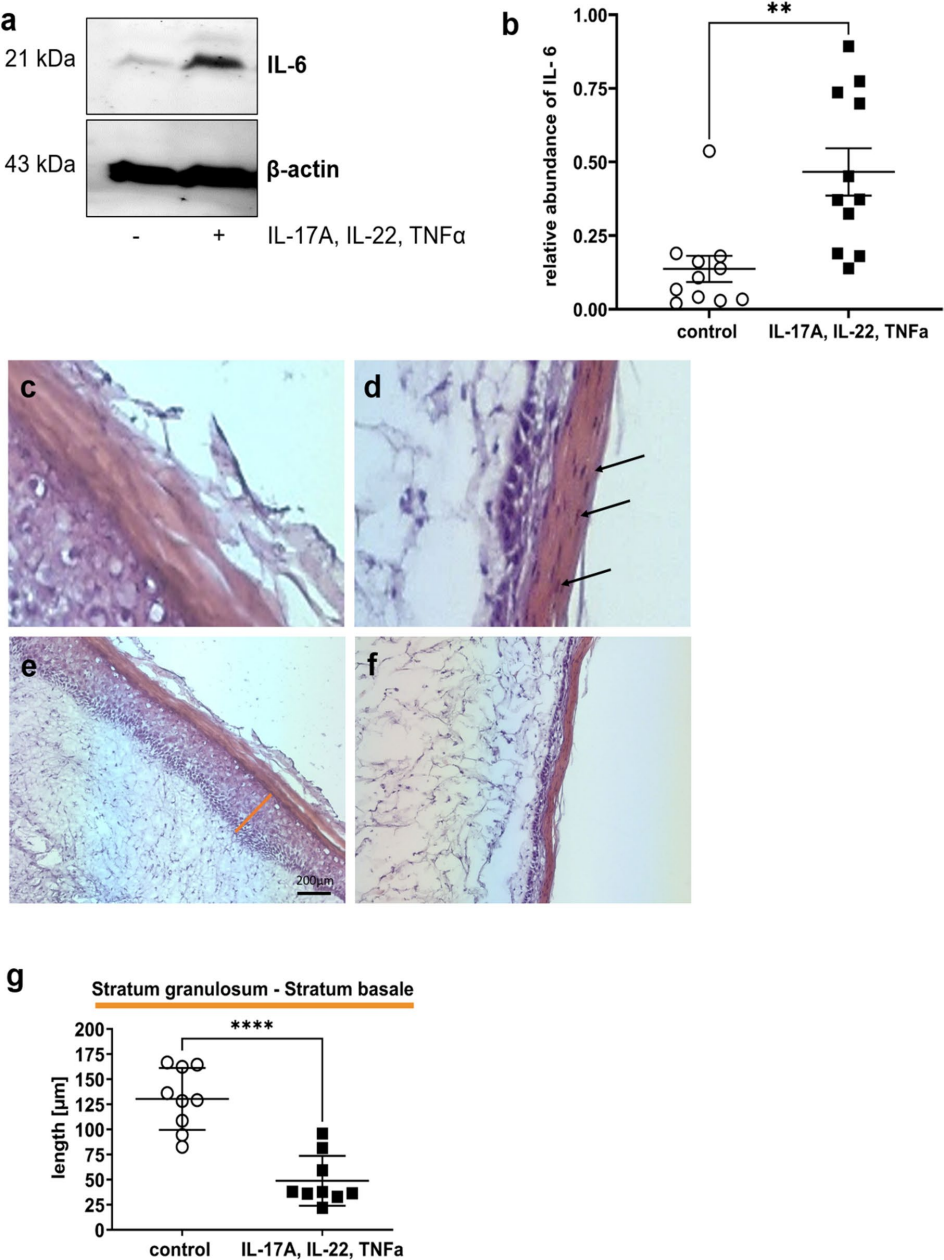
Cytokine-induced psoriasis-like changes in full-thickness skin models. Full-thickness skin models were incubated with cytokines for 5 days at 37 °C. Subsequently, skin models were lysed and submitted to Western blot analysis against IL-6 and β-actin. **a** Western blots from representative experiments are shown (*n* = 11). **b** Diagram depicts densitometric evaluation of IL-6 abundance and adjustment to the corresponding β-actin band. Results are given as arithmetic means ± STABW from eleven independent experiments. Statistical differences were determined by ANOVA and with Bonferroni post hoc test (**p ≤ 0.01). **c** Representative photomicrographs of skin model sections stained with H&E untreated control. **d** The epidermis of the cytokine-treated skin model demonstrates parakeratosis (arrows). **f** Representative images show the H&E staining of the epidermis of untreated control (**e**) versus cytokine-treated skin models. **g** Diagram depicts evaluation of length of the epidermis layers (*stratum basale – stratum granulosum* = orange bar in e). Each specimen was subjected to H&E staining and photographed at a magnification of 200 × . Scale bar, 200 µm. Three images were acquired for each sample. For each skin model, three measurements were taken from different fields of view for each of three individual sections analyzed (9 total measurements/skin model). Results are given as arithmetic means ± STABW. Statistical differences were determined by ANOVA and Bonferroni post hoc test (**p ≤ 0.01; ****p ≤ 0.0001)

In preliminary experiments on diabetic skin which is characterized by dryness, itching, numbness, ulcers, and other pathological changes (Nickel et al. 2021), we observed that C3bot only shows effects on pathologically altered skin (data not shown). Thus, we needed a skin model in which lesions and pathological phenotype, respectively, could be induced for further experiments. Initial experiments were performed to check whether the Phenion^®^ full-thickness skin model is suitable for further experiments with C3bot. First, we demonstrated in Western blot analysis (Fig. 1a) that incubation of the skin model with a cytokine mixture (IL-17A, IL-22, TNF-alpha) led to a significant induction of IL-6 (Fig. 1b). Secondly, incubation of skin model with cytokine mixture induced parakeratosis showed in H&E-stained sections (Fig. 1c and d). Third, after cytokine incubation of the skin model, a change in individual layers of the epidermis was demonstrated (Fig. 1e–g). Histological H&E staining revealed individual layers of the epidermis. The uppermost cornified skin layer, the *stratum corneum*, is stained pink. Below the *stratum corneum*, the *stratum granulosum* can be seen in violet, which merges into the *stratum spinosum*. The *stratum basale* separating the epidermis from the dermis as a cell-rich layer stained dark violet (Fig. 1e). After cytokine treatment of the skin models, it appears that almost only the *stratum basale* is visible under the *stratum corneum* (Fig. 1f). Compared to the control, the skin models treated with cytokines show a significant reduction in the thickness of the *stratum granulosum* to *stratum basale* (Fig. 1g).

All these effects were characteristic for psoriasis, a skin lesion induced by cytokines. Thus, the cytokine mixture was in fact able to transform the original skin model into a skin model of psoriasis.

**Fig. 2:**
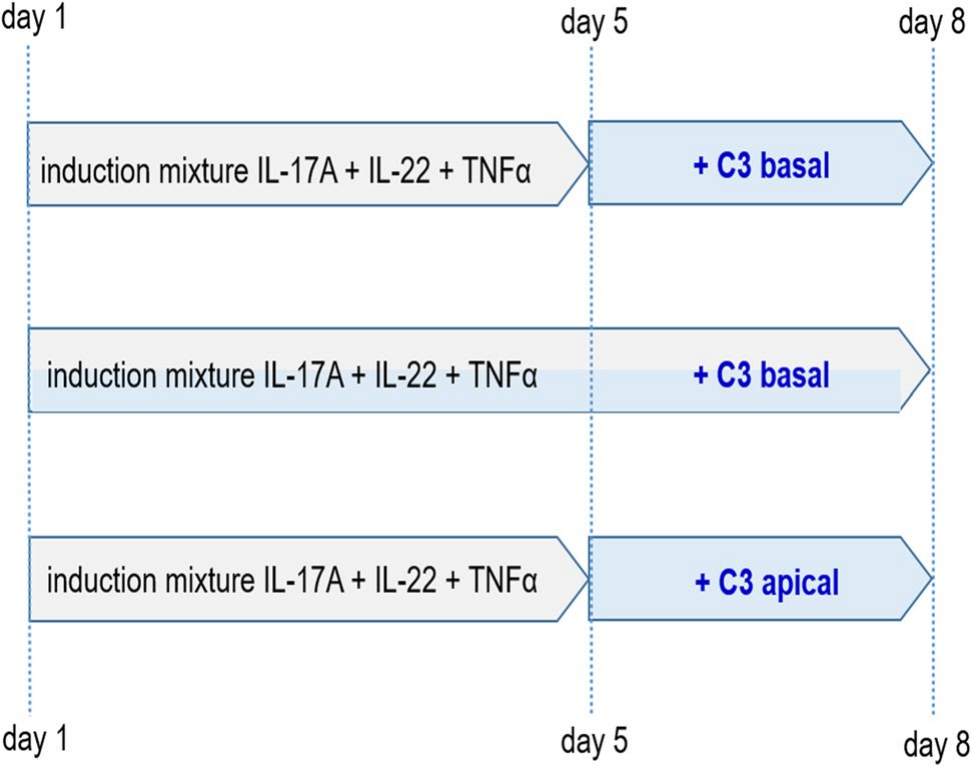
Treatment schemata of the full-thickness skin models. In all three experimental approaches, skin models were incubated with 10 ng/ml IL-17A, 25 ng/ml IL-22, 10 ng/ml TNFα, and 50 nM C3bot as indicated. The medium and additives were changed every 48 h. After the total incubation period, skin models were stringently washed three times, lysed, and submitted to Western blot analysis or H&E staining

To test the assumption that C3bot affects the induced psoriasis-like phenotype, three different experimental approaches were performed (Fig. 2).

In the first set of experiments, the 5-day cytokine-mediated induction of psoriasis phenotype was conducted, followed by a 3-day basal incubation with C3bot. The purpose of this experimental approach was to analyze whether C3bot affected an already existing psoriasis. In the second experimental approach, the skin models were incubated simultaneously with the cytokine mixture and C3bot for 8 days to analyze the effects of C3bot on the development of psoriasis. In the third set of experiments, the 5-day cytokine-mediated induction of psoriasis was performed as in the first experimental approaches. However, C3bot was added apically for 3 days followed by the analysis of the psoriasis-like phenotype.

### Basally applied C3bot reduces cytokine-induced IL-6 expression and partially reverses the cytokine-induced decrease in epidermis thickness in skin model

**Fig. 3:**
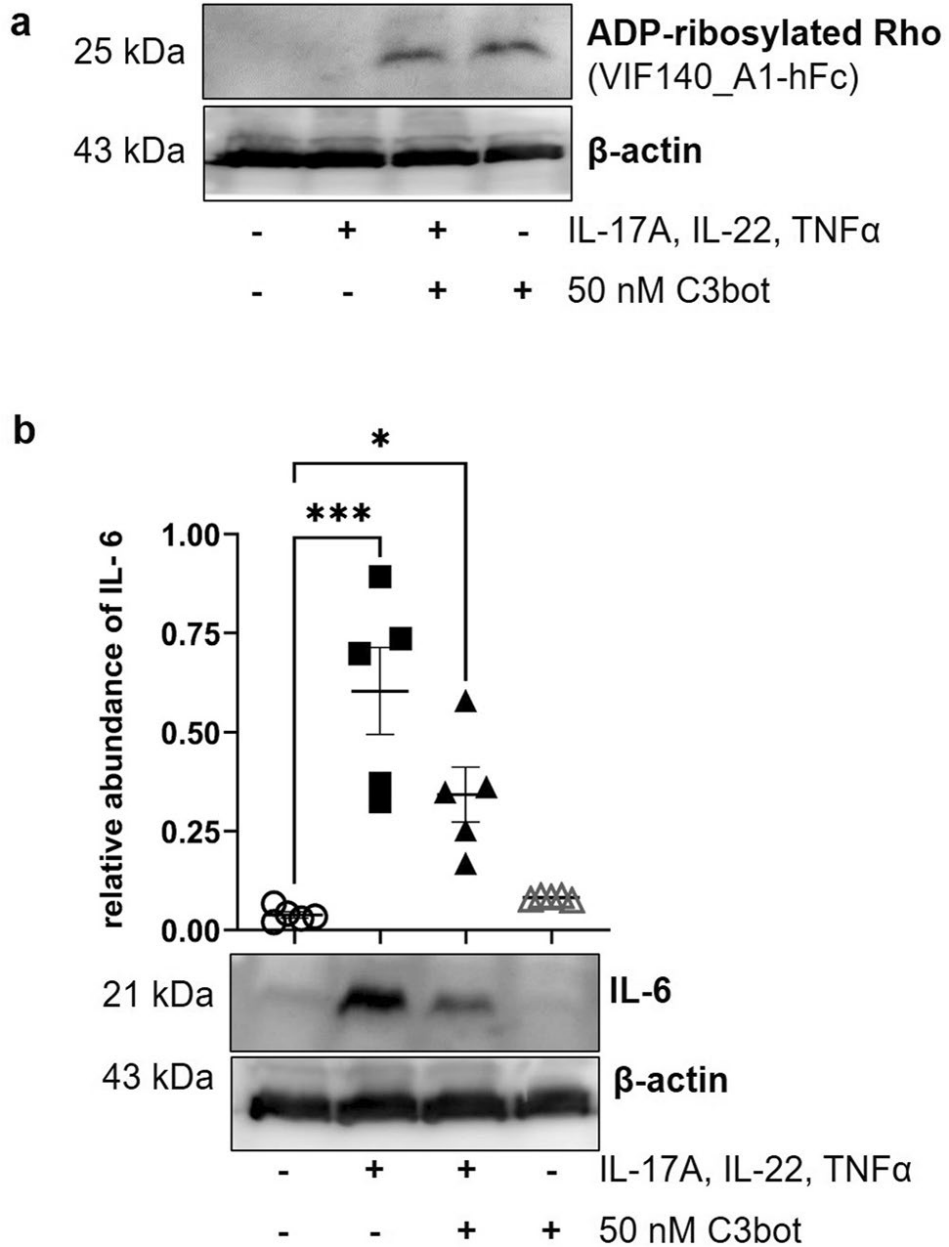
Effects of basally applied C3bot on RhoA and IL-6 proteins. Full-thickness skin models were treated basally with cytokines for 5 days followed by apically C3bot for 3 days as demonstrated in Fig. 2. Subsequently, skin models were stringently washed three times, lysed, and submitted to Western blot analysis against ADP-ribosylated RhoA (**a**) or IL-6 (**b**) and β-actin. Western blots from representative experiments are shown (*n* = 5). **b** Diagram depicts densitometric evaluation of IL-6 abundance and adjustment to the corresponding β-actin band. Statistical differences were determined by ANOVA with Bonferroni post hoc test (*p ≤ 0.05; **p ≤ 0.01; ***p ≤ 0.001)

Skin models were basally incubated with cytokine mixture for 5 days to induce psoriasis-like phenotype. Thereafter, C3bot was basally added for 3 days and then to check whether the psoriasis phenotype including the upregulation of IL-6 is altered. Accessibility of intracellular Rho of the skin model to C3 was demonstrated by ADP-ribosylated RhoA in Western blot analysis (Fig. 3a). Further Western blots showed that the abundance of IL-6 was upregulated by cytokine mixture, as expected for a psoriasis phenotype. The addition of C3bot diminished cytokine-mediated IL-6 abundance (Fig. 3b).

**Fig. 4:**
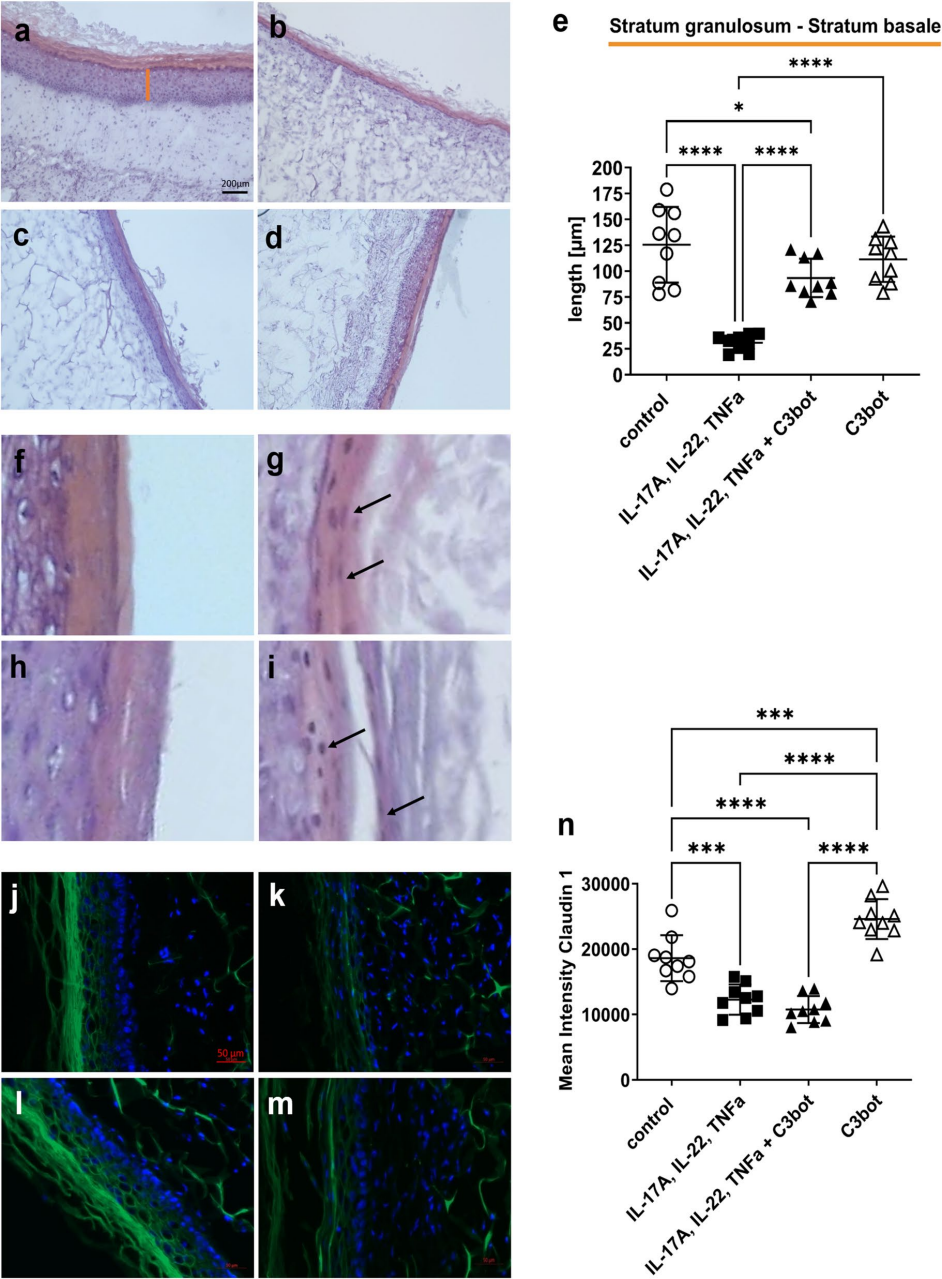
Basally applied C3bot reduces the cytokine-induced decrease in epidermis thickness. Histological findings of treated or untreated full-thickness skin models with hematoxylin and eosin staining, control (**a**), IL-17A, IL-22, TNF-alpha (**b**), 50 nM C3bot (**c**), and IL-17A, IL-22, TNF-alpha + C3bot (**d**). Diagram depicts evaluation of length of the epidermis layers (without *stratum corneum* = orange bar in **a**) (**e**). Each specimen was subjected to H&E staining and photographed at a magnification of 200 × . Scale bar, 200 μm. Statistical differences were determined by ANOVA with Bonferroni post hoc test (*p ≤ 0.05; **p ≤ 0.01; ***p ≤ 0.001; ****p ≤ 0.0001). Representative photomicrographs of cytokine-treated skin models demonstrate parakeratosis (arrows) versus untreated skin models (**f**–**i**). Upper panel: untreated control (**f**) or cytokine pre-treated skin model (**g**), lower panel: C3bot treated skin models (**h**) or cytokine pre-treated skin model + C3bot (**i**). Immunohistochemical analysis of claudin-1 (**j**–**m**). Full-thickness skin models were incubated as shown in Fig. 2. Ten-micrometer tissue sections were prepared and claudin-1 analyzed immunohistochemically. The merged image with the nuclei stained with 4′,6′-diamidino-2-phenylindole (DAPI) (blue) is shown. Results are representative of three separate experiments. Representative photomicrographs show the immunohistochemically localization of claudin-1 (green) in treated or untreated skin models. Scale bar, 50 μm. Control (**j**); IL-17A, IL-22, TNF-alpha (**k**); C3bot (**l**) and IL-17A, IL-22, TNF-alpha + C3bot (**m**). Diagram depicts quantification of claudin-1 abundance (**n**). Statistical differences were determined by ANOVA with Bonferroni post hoc test (*p ≤ 0.05; **p ≤ 0.01; ***p ≤ 0.001; ****p ≤ 0.0001)

In addition, H&E-stained Sects. (10 µm) were prepared, and individual epidermis layers were measured (Fig. 4a). Unfortunately, in some cytokine-treated skin models, the *stratum corneum* detached from the underlying *stratum granulosum* (Fig. 4b), so that an exact measurement of the entire epidermis was not be guaranteed for all skin models. Therefore, the measurement results of the underlying layers *stratum basale* to *stratum granulosum* were used as a stable readout for the thickness of the epidermis. In untreated control (Fig. 4a; 125.5 ± 36.6 µm), *stratum granulosum* to *stratum basale* and *stratum corneum* were separately visualizable. After treatment of skin models with cytokine mixture, *stratum corneum* became significantly narrower/thinner. The *stratum granulosum* and *stratum basale* were also significantly reduced (31 ± 7.7 µm) (Fig. 4b). After addition of C3bot (Fig. 4d), the cytokine-induced decrease in *stratum granulosum* to *stratum basale* layer was significantly smaller (93.4 ± 18.6 µm), i.e., C3bot counteracted the cytokine effect but did not reverse it completely. The incubation of original skin models with C3bot (Fig. 4c) caused a moderate decrease in the thickness of the epidermis layer (C3bot 111.5 ± 22 µm). As mentioned earlier, parakeratosis is a marker of psoriasis-like phenotype and was analyzed in this experimental approach (Fig. 4f–i). Parakeratosis was observed in all cytokine-incubated skin models (Fig. 4g and i). Unfortunately, basal addition of C3bot had no effect on parakeratosis (Fig. 4i). Immunohistochemically analysis of claudin-1 (Fig. 4j–m) showed that the transformation of skin model to the psoriasis phenotype by cytokine mixture treatment led to a significant decrease in the total claudin-1 signal (Fig. 4k). Addition of C3bot (Fig. 4m) did not alter the cytokine-induced effect on the reduced claudin-1 abundance. However, C3bot alone seemed to increase claudin-1 signaling (Fig. 4n).

The data showed that basally applied C3bot reduced the cytokine-mediated decrease in epidermis thickness, which was thought to be due to inhibition of RhoA. Nevertheless, the results suggest that basally applied C3bot had only minor effects on the psoriasis-like phenotype despite inhibition of RhoA and reduction of IL-6 abundance in induced psoriasis.

### Continuous presence of C3bot at the basal side prevents cytokine-mixture-induced IL-6 expression but not the cytokine-induced psoriasis-like morphological changes

**Fig. 5:**
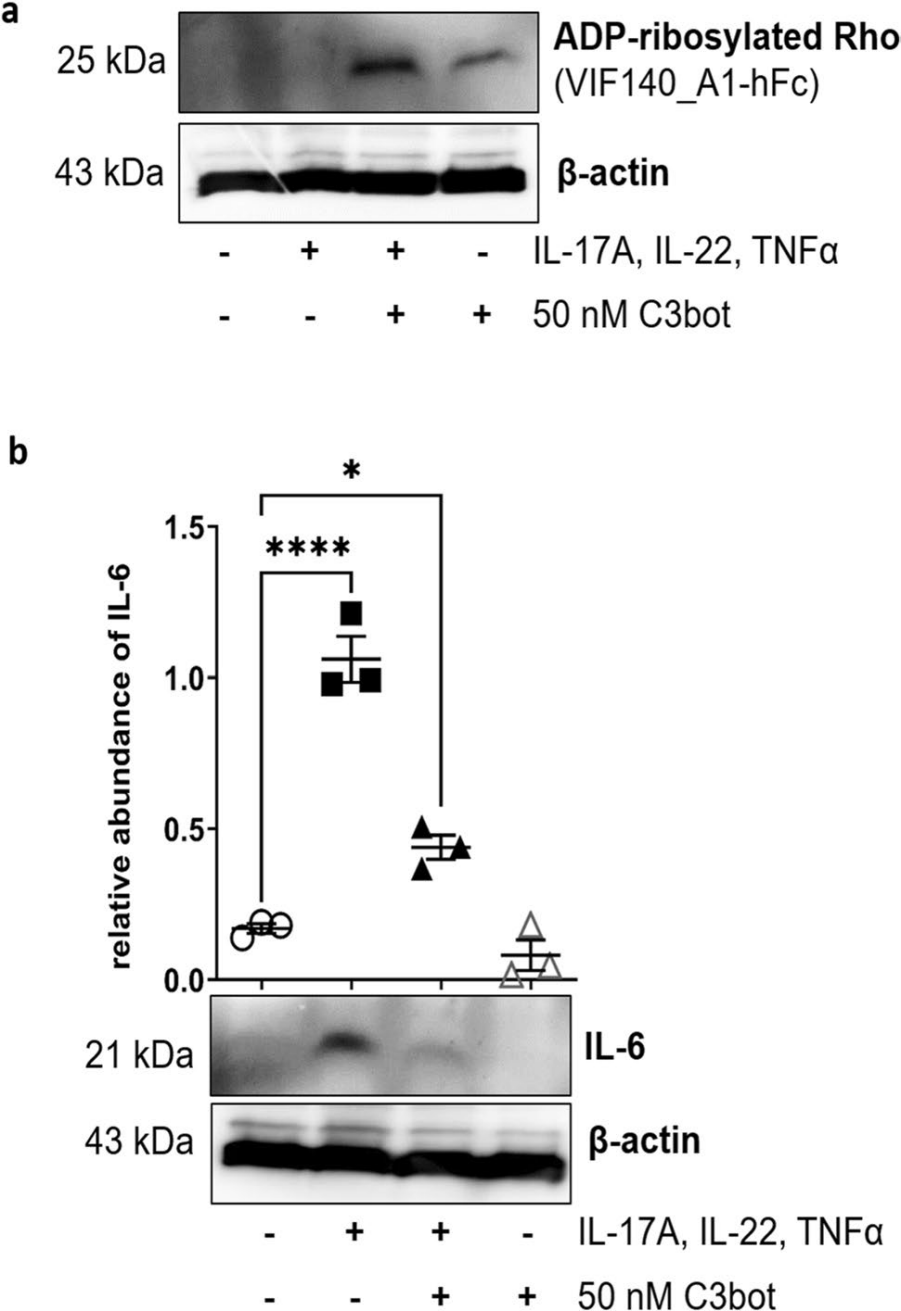
Continuous presence of C3bot at the basal side prevents cytokine-induced IL-6 expression. Full-thickness skin models were basally incubated with cytokines and simultaneously with C3bot for 8 days as demonstrated in Fig. 2. Subsequently, skin models were stringently washed three times, lysed, and submitted to Western blot analysis against ADP-ribosylated RhoA (**a**) or IL-6 (**b**) and β-actin. Western blots from representative experiments are shown (*n* = 3). **b** Diagram depicts densitometric evaluation of IL-6 abundance and adjustment to the corresponding β-actin band. Statistical differences were determined by ANOVA and Bonferroni post hoc test (**p ≤ 0.01; ****p ≤ 0.0001)

In this part of the study, skin models were basally incubated with cytokine mixture and simultaneously with C3bot for 8 days to record the cytokine-induced transformation to the psoriasis-like phenotype (Fig. 2). Under this experimental condition, C3bot entered the cells of skin model as proved by Western blot analysis of ADP-ribosylated RhoA (Fig. 5a). A cytokine-induced upregulation of IL-6 was also detected. Incubation of skin models with C3bot in presence of cytokine mixture led to a strong reduction in IL-6 abundance (Fig. 5b). The reduction in IL-6 abundance was much more pronounced than in the first experimental approach, where C3bot was applied after manifestation of psoriasis phenotype.

**Fig. 6:**
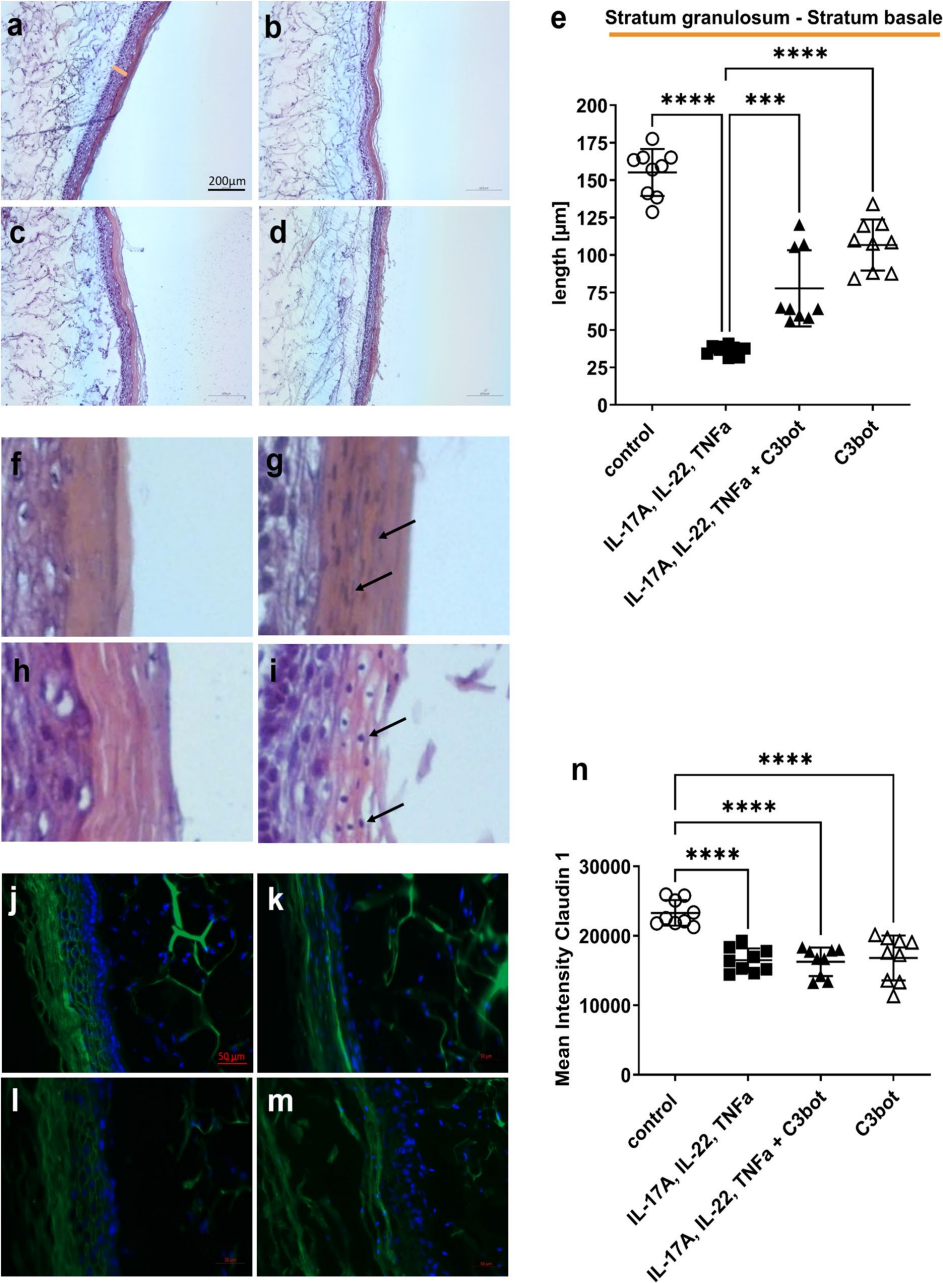
Continuous presence of C3bot at the basal side has no effect on the cytokine-induced psoriasis-like morphological changes. Histological findings of treated or untreated full-thickness skin models with hematoxylin and eosin staining, control (**a**), IL-17A, IL-22, TNF-alpha (**b**), 50 nM C3bot (**c**), and IL-17A, IL-22, TNF-alpha + C3bot (**d**). Diagram depicts evaluation of length of the epidermis layers (without *stratum corneum* = orange bar in **a**) (**e**). Each specimen was subjected to H&E staining and photographed at a magnification of 200 × . Scale bar, 200 µm. Statistical differences were determined by ANOVA with Bonferroni post hoc test (*p ≤ 0.05; **p ≤ 0.01; ***p ≤ 0.001; ****p ≤ 0.0001). Representative images of cytokine-treated skin models demonstrate parakeratosis (arrows) (**f**–**i**). Upper panel: untreated control (**f**) or cytokine pre-treated skin model (**g**), lower panel: C3bot treated skin model (**h**) or cytokine pre-treated skin model and C3bot (**i**). Immunohistochemical analysis of claudin-1 (**j**–**m**). Full-thickness skin models were incubated as shown in Fig. 2 and stained using immunohistochemically methods with an antibody against claudin-1 and DAPI (blue, cell nuclei). Representative images of claudin-1 and DAPI nuclear staining. Scale bar, 50 µm. Control (**j**); IL-17A, IL-22, TNF-alpha (**k**); C3bot (**l**) and IL-17A, IL-22, TNF-alpha + C3bot (**m**). Diagram depicts quantification of claudin-1 abundance (**n**). Statistical differences were determined by ANOVA with Bonferroni post hoc test (*p ≤ 0.05; ****p ≤ 0.0001)

Again, H&E-stained sections were performed from the skin models, and epidermal layer was measured (Fig. 6a–d). The basal incubation with cytokines (Fig. 6b) caused a significant decrease in the epidermal layer (36.8 ± 3.4 µm), comparable to the results of previous experiments. The addition of C3bot (Fig. 6d) again led to an attenuation of the cytokine-induced effect on epidermal layer thickness (*stratum granulosum* to *stratum basale*, 77.8 ± 25.4 µm). Noteworthy, skin models incubated alone with C3bot for 8 days also showed a significant reduction in *stratum granulosum* to *stratum basale* (C3bot 106.7 ± 17.1 µm) compared to untreated control (155.1 ± 15.7 µm) (Fig. 6e). This result suggested a RhoA-dependent effect. Also in these experimental approaches, parakeratosis was detected in all cytokine-treated skin models, and the addition of C3bot did not affect parakeratosis (Fig. 6f–i). Immunohistochemically analysis of claudin-1 (Fig. 6j–m) revealed that incubation with cytokines for 8 days led again to a significant decrease of the total claudin-1 signal (Fig. 6k and m). Simultaneous incubation with C3bot did not cause any additional drop in claudin-1 signal (Fig. 6n).

However, incubating skin models with C3bot alone resulted in a partly strong decrease of claudin-1 signaling and suggests a consequence of C3bot-mediated RhoA inhibition. In summary, the data show that simultaneous incubation of the skin models with cytokine mixture and C3bot led to an almost complete inhibition of IL-6 abundance. However, this did not seem to have any effect on the parameters we analyzed, since we only observed small changes in epidermis thickness and none in parakeratosis or claudin-1 signal.

### Apically applied C3bot decreases cytokine-induced IL-6 expression and amplifies the cytokine-induced depletion of epidermis thickness

**Fig. 7:**
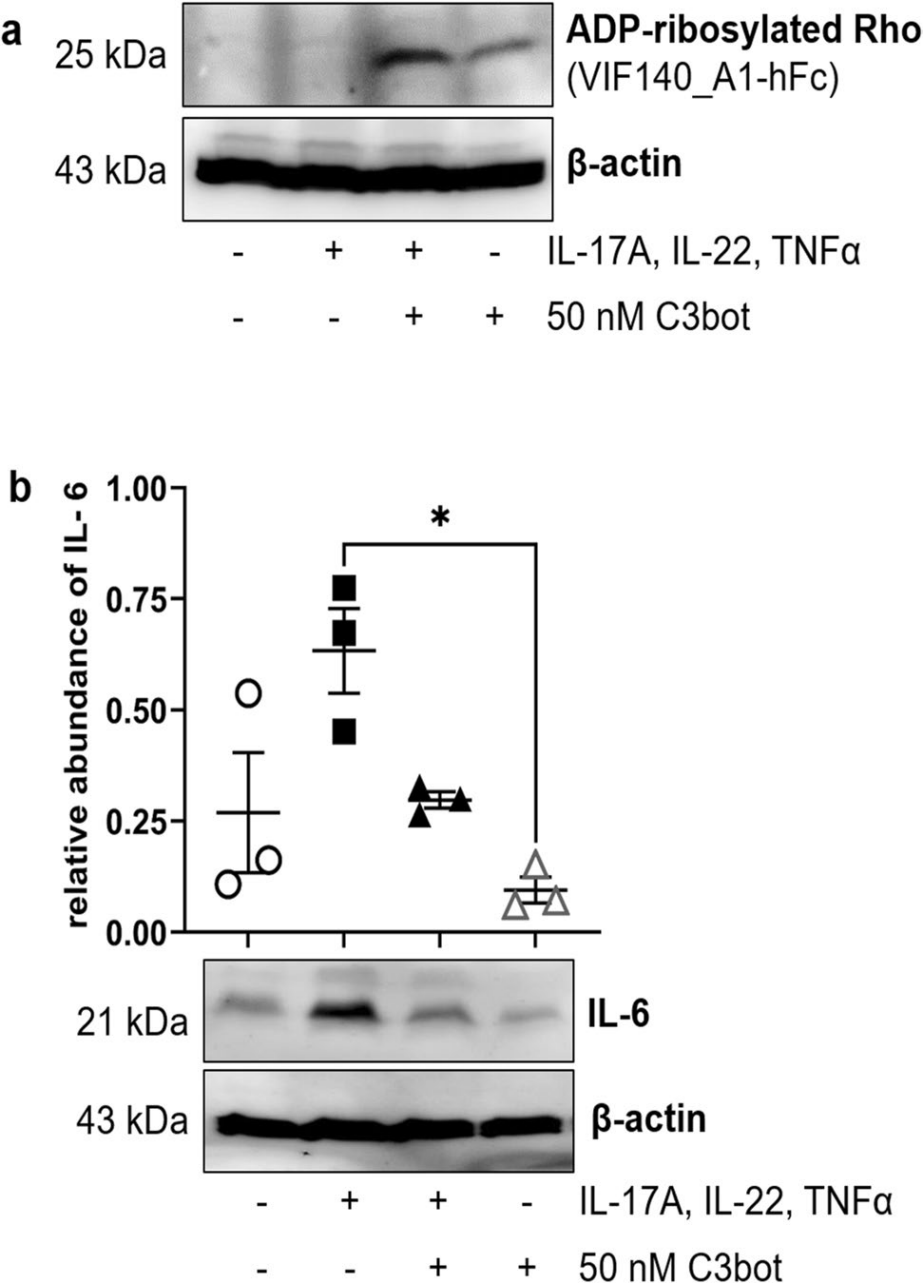
Apically applied C3bot ADP-ribosylates intracellular RhoA and decreases cytokine-induced IL-6 expression. Full-thickness skin models were treated basally with cytokines for 5 days followed by apically C3bot for 3 days, as demonstrated in Fig. 2. Subsequently, skin models were stringently washed three times, lysed, and submitted to Western blot analysis against ADP-ribosylated RhoA (**a**) or IL-6 (**b**) and β-actin. Western blots from representative experiments are shown (*n* = 3). Diagram depicts densitometric evaluation of IL-6 abundance and adjustment to the corresponding β-actin band (**b**). Statistical differences were determined by ANOVA and Bonferroni post hoc test (*p ≤ 0.05)

In the third part of the study, skin models were basally incubated with cytokines for 5 days to induce psoriasis. Thereafter, C3bot was apically added for 3 days (Fig. 2). ADP-ribosylated RhoA was detected in Western blot analysis and proved intracellularly active C3bot taken up via the *stratum corneum* (Fig. 7a). A cytokine-induced upregulation of IL-6 was also detected (Fig. 7b). Furthermore, apical application of C3bot reduced IL-6 abundance indicating RhoA dependence of the reduction of IL-6 abundance.

**Fig. 8:**
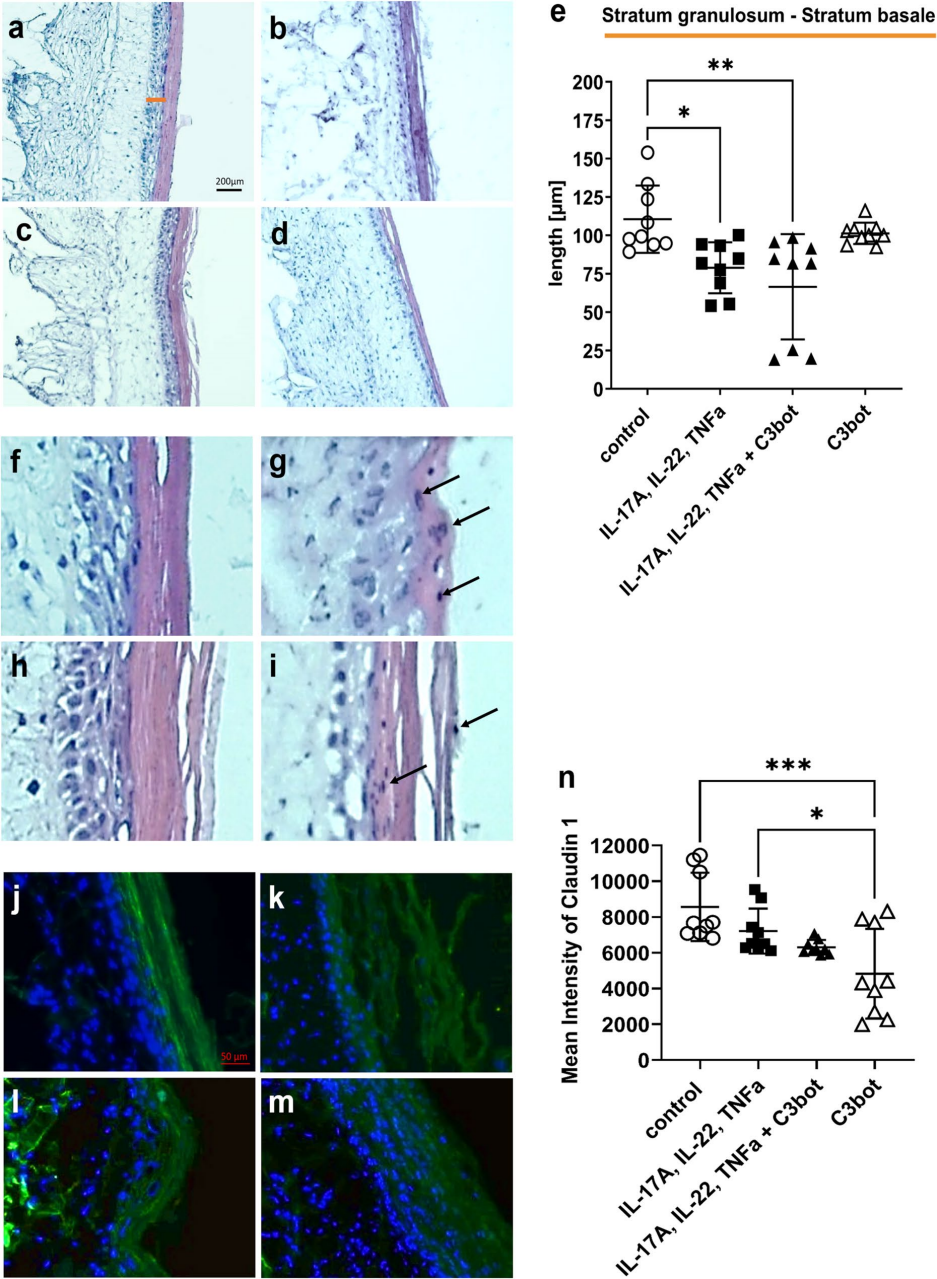
Apically applied C3bot amplifies the cytokine-induced depletion of epidermis thickness. Histological findings of treated or untreated full-thickness skin models with hematoxylin and eosin staining, control with 1% DMSO (**a**), IL-17A, IL-22, TNF-alpha with 1% DMSO (**b**), 50 nM C3bot with 1% DMSO (**c**), and IL-17A, IL-22, TNF-alpha + C3bot with 1% DMSO (**d**). Diagram depicts quantification of epidermis thickness (without *stratum corneum* = orange bar in a) (**e**). Each specimen was subjected to H&E staining and photographed at a magnification of 200 × . Scale bar, 200 µm. Statistical differences were determined by ANOVA with Bonferroni post hoc test (*p ≤ 0.05; **p ≤ 0.01). Representative images of cytokine-treated skin models demonstrate parakeratosis (arrows) (**f**–**i**). Upper panel: untreated control (**f**) or cytokine pre-treated skin model (**g**), lower panel: C3bot treated skin model (**h**) or cytokine pre-treated skin model and C3bot (**i**). Immunohistochemical analysis of claudin-1(**j**–**m**). Full-thickness skin models were incubated as shown in Fig. 2 and stained using immunohistochemically methods with an antibody against claudin-1 and DAPI (blue, cell nuclei). Representative images of claudin-1 and DAPI nuclear staining. Scale bar, 50 μm. Control (**j**); IL-17A, IL-22, TNF-alpha (**k**); C3bot (**l**), and IL-17A, IL-22, TNF-alpha + C3bot (**m**). Diagram depicts quantification of claudin-1 abundance (**n**). Statistical differences were determined by ANOVA and Bonferroni post hoc test (*p ≤ 0.05; **p ≤ 0.01; ***p ≤ 0.001)

As before, basal incubation of skin models with cytokines led to a clear reduction in *stratum granulosum* to *stratum basale* layer (untreated 110.5 ± 21.9 µm; cytokine treated 78.9 ± 16.6 µm) (Fig. 8a–d). Interestingly, additional apical addition of C3bot (66.5 ± 34.3 µm) resulted in a more pronounced decrease in layer thickness (Fig. 8d). The exclusive apical incubation of the skin models with C3bot caused only a moderate reduction in the layer thickness (C3bot 101.3 ± 6.9 µm) (Fig. 8c). In general, it had to be mentioned that the data set is heterogeneous (Fig. 8e). As in previous experimental approaches, an apically application of C3bot did not reduce or even prevent the development of parakeratosis (Fig. 8f–i), so that a clear parakeratosis was shown in all skin models that were incubated with cytokines (Fig. 8g and i). Immunohistochemically analysis showed a decrease in signal of claudin-1 after cytokine incubation (Fig. 8i–m) and a significant decrease in claudin-1 signal after apical addition of C3bot (Fig. 8l and m). The sole apical addition of C3bot constructs also caused a strong decrease in the signal (Fig. 8n).

The results show that apically C3bot was taken up into the keratinocytes of the *stratum corneum* and led to both ADP-ribosylation of RhoA and inhibition of IL-6 abundance. But even in this experimental approach, C3bot did not seem to have a positive effect on the psoriasis-like phenotype. On the contrary, C3bot even enhanced the cytokine-mediated decrease in epidermal thickness.

## Discussion

In the present study, the potential of C3bot to modulate the psoriasis-like phenotype was assessed, with different experimental approaches, using the commercially available Phenion^®^ full-thickness skin model. This skin model consists of epidermal and dermal skin compartments, whereas the keratinocytes proliferate on the dermal equivalent consisting of fibroblast cells immersed in a collagen gel. Reconstructed skin models can be modulated by adding different cell culture media or cytokines. Bernard et al. hypothesize that cytokines such as IL-17, IL-22, and TNF-alpha are responsible for the development of psoriasis phenotypes. Indeed, treatment of the reconstructed human epidermis with a mixture of these cytokines resulted in a psoriasis phenotype (Bernard et al. 2012). The psoriasis-like phenotype in the skin model is characterized, among other things, by increased production of IL-6 (Grossman et al. 1989), parakeratosis, and dysregulation of the epidermal layers (Todorović et al. 2022). In fact, our results demonstrated that incubation of the skin models with the cytokine mixture leads to a variety of phenotypic features (like IL-6 expression, parakeratosis, epidermal changes) similar to psoriatic skin lesions and confirmed the usefulness of the cytokine-stimulated Phenion^®^ full-thickness skin model for further investigations with C3bot.

In all experimental approaches, fibroblasts and keratinocytes stimulated by pro-inflammatory cytokines (IL-17, IL-22, and TNF-alpha), induced production of IL-6, its increase is differentially modulated by C3bot (Table 1).

**Table 1:** Summary table of results

Measured items	No C3	C3bot		
		Basal	Simultaneous	Apical
ADP-ribosylated RhoA	-	** + **	** + **	** + **
IL-6 abundance	↑↑↑	↑	↑	↑↑
Epidermis thickness	↓↓↓	↓	↓↓	↓↓↓↓
Claudin-1 signal	↓	↓↓	↓	↓↓

This table shows the results of C3bot used after pre-↑incubation of the skin models with the cytokine mix. The skin models were treated as shown in Fig. 2. In all experimental approaches, skin models were incubated with 10 ng/ml IL-17A, 25 ng/ml IL-22, 10 ng/ml TNFα, and 50 nM of C3bot. Subsequently, Western blot analysis of ADP-ribosylated RhoA and IL-6 was performed. Furthermore, 10-µm cryostat sections were prepared of all skin models and H&E stained, and the thickness of the epidermis was determined. The immunohistochemical detection of claudin-1 and the evaluation of the signal intensity were carried out. IL-6 abundance: ↑ strong signal, ↑↑ stronger signal, ↑↑↑ strongest signal; epidermal thickness: ↓ slight decrease, ↓↓ significant decrease, ↓↓↓ sharp decrease, ↓↓↓↓ very sharp decrease; claudin-1 signal: ↓ weak signal, ↓↓ weaker signal

The results suggest that the expression of IL-6 is regulated by RhoA as its inhibition by C3 has profound implications. Previous studies indicated that Rho GTPases play a role in the expression and secretion of IL-6. Kang and co-worker reported that Rho-kinase inhibitor Y-27632 downregulates LPS-induced IL-6 in human gingival fibroblasts (Kang et al. 2018). The involvement of Rho/Rho-kinase signaling pathway in IL-6 secretion was also described in trophoblast (Goyal et al. 2013). Interleukin-6 is a multifunctional cytokine and plays a major role in the regulation of immune responses. IL-6 is involved in the healing of cutaneous wounds but also induces the release of pro-inflammatory cytokines from tissue resident macrophages, keratinocytes, endothelial cells, and stromal cells (Johnson et al. 2020). Dysregulation of IL-6 can lead to skin pathology like fibrosis or psoriasis (Grossman et al. 1989; Lowes et al. 2007). It is well-documented that both fibroblasts and keratinocytes produce IL-6 when stimulated but fibroblasts more rapidly form larger amounts of IL-6 than keratinocytes do (Waelti et al. 1992; Boxman et al. 1995; Ben Abdallah et al. 2021). Therefore, we are convinced that in the application of the full-thickness skin model fibroblasts rather than keratinocytes harbors the ability to produce higher levels of IL-6. IL-6 stimulates the keratinocytes to proliferate and produce further inflammatory cytokines, which recruit and further stimulate immune cells and drive the psoriatic inflammatory process. When C3bot is basally added to the skin model, IL-6 production in the fibroblasts is inhibited to a greater extent, and the IL-6-mediated effects on the keratinocytes are thus modulated to a greater extent. When C3bot is apically added to the full-thickness skin model, IL-6 production by fibroblasts is only slightly inhibited, and the IL-6-mediated effects on the keratinocytes are therefore hardly modulated. The results indicate a cell-specific difference in the C3bot effect, with the fibroblasts reacting more sensitively to the C3bot than the keratinocytes do.

By the way, in all three experimental settings, ADP-ribosylated RhoA was detected in Western blot analysis, even when the skin models were apically treated with C3bot. Notably, the *stratum corneum* limits dermal and transdermal drug delivery and acts as the main barrier for most compounds or drugs. Despite these cellular limitations, C3bot enters the keratinocytes of the *stratum corneum* demonstrating again that C3bot is taken up in a variety of cells and not just specifically in macrophages.

Despite the heterogeneous histological data, C3bot modulates the effects of cytokine-induced psoriasis-like phenotype. Basally applied C3bot decreases the extent of cytokine-induced depletion of *stratum granulosum* to *stratum basale* layer, whether added after cytokine-mediated psoriasis induction or simultaneously with cytokines. In contrary, apically added C3bot reveals a stronger decrease in *stratum granulosum* to *stratum basale* thickness. In all three experimental approaches, the exclusive incubation of skin models with C3bot leads to significant changes in layer thickness of *stratum granulosum* to *stratum basale*. These results indicate a RhoA-dependent effect.

Many studies show that full epidermal thickness was increased in psoriasis patients compared to controls based on enhanced proliferation and abnormal differentiation of keratinocytes (Alper et al. 2004; Odrzywołek et al. 2022). However, plaque-type psoriasis, the most common subtype of psoriasis, can be divided into further subtypes such as thin and thick plaques based on their epidermal thickness (Christensen et al. 2006). Recently, histological measurements of epidermal thickness on an image of skin biopsy tissue confirmed a mixture of two subpopulations—thick and thin plaque psoriasis (Kim et al. 2015). In general, psoriasis manifests itself as a heterogeneous phenotype with several subtypes, but in all clinical studies, an increase in epidermal thickness compared to controls was demonstrated. In contrast to these studies, our results showed that full epidermal thickness were decreased in the cytokine-induced psoriasis skin model compared to untreated controls. Recently published data demonstrated that treatment of full-thickness skin model with cytokine mixture leads to a decrease in the thickness of the nucleated epidermis (Todorović et al. 2022). In a long-term skin model with nanometer-thick fibronectin–gelatin-coated normal human dermal fibroblasts, a 5-day incubation with rhIL-17A causes a decrease in the thickness of the *stratum granulosum* (Singh et al. 2020). Moreover, higher concentrations of cytokine mixture result in tissue disruption and detachment of the *stratum corneum* (Todorović et al. 2022). We also observed detachment of the *stratum corneum* in some skin models treated with both cytokines and C3bot. While these results support our findings, they differ from observations on human skin biopsies as mentioned above. Further studies are needed to clarify this discrepancy in detail.

The effect of accelerated migration of keratinocytes in psoriasis is the parakeratosis, showing the presence of nuclei in the cells of the *stratum corneum*. In our study, we detect cell nuclei in the *stratum corneum* and thus parakeratosis in all skin models that have been treated with cytokines. Neither the basally nor the apically addition of C3bot has an effect on parakeratosis. These results suggest that RhoA has no impact on the development of parakeratosis and that the accelerated migration of keratinocytes is regulated independently of Rho signaling. The role of RhoA in migration is less clear and might be cell-type dependent. For keratinocytes, however, the data situation is not clear. RhoA-deficient keratinocytes showed a reduced migration speed and an increased tortuosity. Surprisingly, treatment with the ROCK inhibitor Y27632 increased migration speed and decreased tortuosity, both in control and in RhoA-deficient keratinocytes. The Jackson group demonstrated that RhoA is dispensable for skin development and maintenance (Jackson et al. 2011).

Another important characteristic of psoriasis is an alteration of tight junction proteins. Tight junctions are located at the most apical part of the epithelial junctional complexes and consist of claudin and occludin. Tight junctions construct epithelial cellular sheets and participate in the polarity of epithelial cells through their own polymerization (Tsukita and Furuse 1999). In the skin, tight junctions are key contributors to the epidermal paracellular barrier, and claudin-1, a main component of tight junctions in the epidermis, is reported to be indispensable for this barrier function. Abnormalities in claudin-1 cause human skin diseases and play a key role in chronic inflammatory skin disease (Hadj-Rabia et al. 2004; De Benedetto et al. 2011). Additionally, altered localization of claudin-1 in the epidermis was described in plaque-type psoriasis (Watson et al. 2007). Claudin-1 that are normally found in all layers of the epidermis was observed at decreased levels, especially in the lowermost *(stratum basale)* but also in the uppermost layers *(stratum corneum)* of the epidermis in early-stage psoriasis (Kirschner et al. 2009). The loss of claudin-1 might reflect an impairment of tight junction barrier function at this stage of psoriatic skin disease. Altered expression of claudin-1 in psoriasis is presumably due to rapid turnover and impaired differentiation of keratinocytes in the upper epidermis. It has long been known that claudin-1 is concentrated in the *stratum granulosum* (Sugawara et al. 2013). However, recently, Haftek et al. reported that claudin-1 is also localized in the *stratum corneum* of the epidermis (Haftek et al. 2022). Moreover, claudin-1 is part of a highly dynamic system of tight junction assembly and can change its local distribution. At the cellular level, it has already been described that claudin-1 relocalized from an apical site to apical, lateral, and basolateral surfaces of Caco-2 cells (Mee et al. 2008). In all three experimental approaches of our study, claudin-1 was observed in the *stratum granulosum* at the cell periphery but also diffusely in lamellar structures in the *stratum corneum*. C3bot did not abolish the cytokine-induced reduction in claudin-1 signal. On the contrary, apically added C3bot even enhanced the cytokine-induced decrease in claudin-1 signal. Next, altered localization and formation of zonula occludens-1 in the epidermis have been described in plaque-type psoriasis. Zonula occludens-1 was expressed in the granular cell layer in the psoriatic perilesional epidermis. In the psoriasis plaques, zonula occludens-1 were detected in a wider zone extending from the granular layer to the middle spinous cell layers (Peltonen et al. 2007). Detailed investigation of our skin models revealed that C3bot has no effect on the zonula occludens-1 signal (data not shown).

The few effects observed in the tight junction can perhaps be attributed to the use of a skin model, which of course also has limitations. Limitations of cytokine-induced psoriasis skin model include the absence of blood vessels, immune cells, and microenvironments. Additionally, the used skin model is limited by skin donor availability and variability. Nevertheless, the used full-thickness skin models are useful for investigating the molecular mechanism of the disease, including keratinocyte differentiation and response to stimuli. In addition, psoriasis is a very complex disease involving multiple interactions between different cell types, thus cell lines or cultured keratinocytes are in fact not suitable for examination of psoriasis pathology or anti-psoriasis drugs. However, an ideal psoriasis model should reflect the clinical hallmarks of human psoriasis, including characteristic histomorphological features, and similar pathogenesis, and should respond similarly to therapeutic agents.

To summarize our results, we showed that incubation of full-thickness skin models with cytokines leads to a psoriasis-like phenotype with reduced epidermis thickness, parakeratosis, and IL-6 induction. Furthermore, our results demonstrated that the expression and localization of tight junction proteins like claudin-1 are not influenced by C3bot. However, an importing finding is that the application of C3bot counteracts cytokine-mediated IL-6 induction, which further supports an involvement of Rho in the pro-inflammatory pathway.

## Data Availability

The datasets generated during and/or analyzed during the current study are available from the corresponding author on reasonable request.

## Abbreviations

C3bot: C3 exoenzyme derived from *Clostridium botulinum*
SDS: Sodium dodecyl sulfate
PAGE: Polyacrylamide gel electrophoresis
IL-6: Interleukin-6
